# Cerebral embolism following thrombolytic therapy for acute myocardial infarction: the second reported case

**Published:** 2010-06

**Authors:** MEHMET BOSTAN, AYH AN KANAT, MURAT SEN, IR KAZDAL, BOSTAN HABIB

**Affiliations:** Department of Cardiology, Rize University Medical School, Rize, Turkey; Department of Neurosurgery, Rize University Medical School, Rize, Turkey; Department of Neurology, Education and Research Hospital, Rize, Turkey; Department of Anesthesiology and Reanimation, Rize University Medical School, Rize, Turkey; Department of Anesthesiology and Reanimation, Rize University Medical School, Rize, Turkey

**Keywords:** acute myocardial infarction, thrombolytic therapy, cerebral emboli

## Abstract

ST-elevation myocardial infarction (STEMI), caused by acute occlusion of the infarct-related coronary artery, is an emergency condition. The primary therapy is restoration of full antegrade flow by either percutaneus coronary intervention (PCI) or thrombolytic therapy (TT). Although primary PCI is superior to TT in patients with STEMI, there are many limitations in clinical practice. TT decreases mortality in STEMI patients, but as experience with thrombolytic agents grows, the potential risks of serious side effects become more apparent. The major complications are bleeding, hypotension and skin rash.

We report on a case of cerebrovascular accident (CVA) caused by cerebral emboli following TT. We concluded that the fact that the patient was in arterial fibrillation (AF) was a major contributing factor to her CVA. This is an extremely rare condition, and our case appears to be the second one reported on in the literature.

## Introduction

One case of cerebrovascular accident due to cerebral emboli following thrombolytic therapy (TT) has previously been published.^1^ ST-elevation myocardial infarction (STEMI) is caused by complete occlusion of the coronary artery. The goldstandard therapy for STEMI is recanalisation of the infarctrelated coronary artery as soon as possible by pharmacological or mechanical means.^2^ Indications for this are STEMI or new left bundle branch block (LBBB) presenting within 12 hours of the onset of symptoms.^2^

Primary percutaneus coronary intervention (PCI) is superior to TT but there are many limitations to PCI. TT is therefore an effective treatment of choice in STEMI, as it is easy to perform anywhere and at any time.

All thrombolytic agents share a common mechanism of activating plasminogen into plasmin, which in turn activates the fibrin degradation pathway. The efficacy and safety of various thrombolytic agents have been well documented in large clinical trials.^3^-^5^

The most feared complication of fibrinolytic treatment is intracranial haemorrhage. Risk factors for haemorrhagic complications include increasing age, elevated pulse pressure, uncontrolled hypertension, recent stroke or surgery, the presence of a bleeding diathesis, and severe congestive heart failure. Although the haemorrhagic complications of TT are well documented, this type of embolic cerebral infarction has not been documented in the literature. Hence we report on our case.

## Case report

**Fig. 1.**
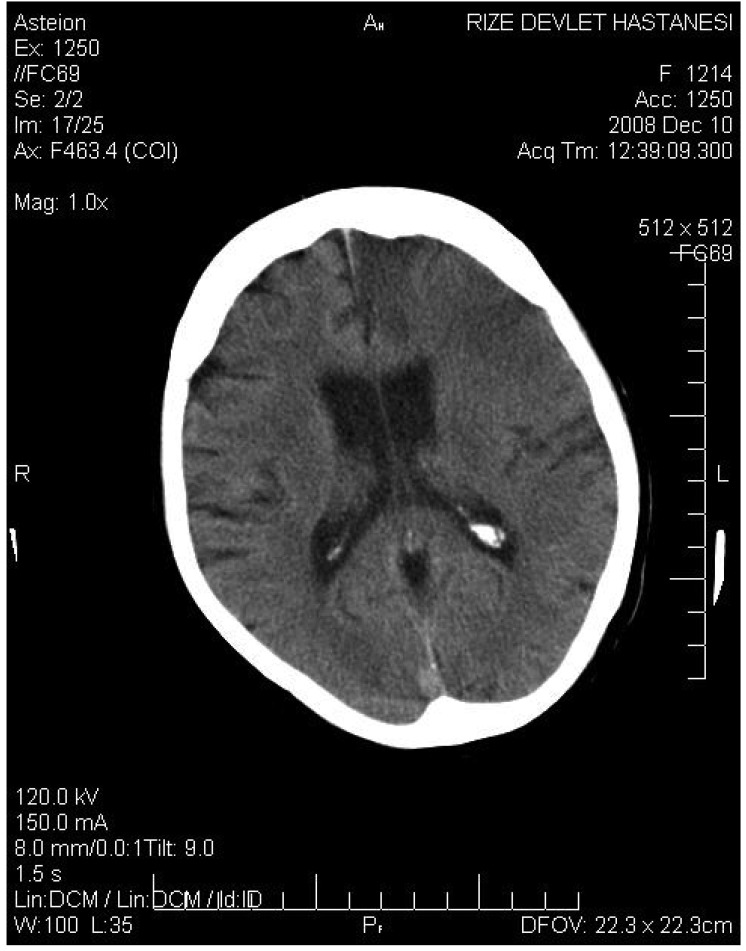
Electrocardiography showing atrial fibrillation and ST -segment elevation in leads D2, D3 and AV F.

An 80-year-old female patient presented to the emergency department with severe chest pain. The duration between the onset of pain and presentation to hospital was 60 minutes. Electrocardiography revealed atrial fibrillation (AF) and ST-segment elevation in leads D2, D3 and AVF (Fig. 1). Physical examination of the patient in the orthopneic position revealed shortness of breath and her blood pressure was 135/83 mmHg. Primary PCI would not therefore have been appropriate for this patient and we decided to use TT.

Laboratory findings were as follows: creatinine kinase-MB: 25 ng/ml, troponin I: 18 ng/ml, and normal liver function tests. The patient had been seeing a cardiologist (MB) with regular outpatient visits. The patient had left ventricular (LV) failure and the last cardiac echocardiograph had been performed one month earlier. In that evaluation, no trombus was detected. The chest pain was very severe on admission, but a new echocardiograph could not be done before TT. Her orthopnoeic breathing was assumed to be due to her LV failure.

Acute inferior myocardial infarction was diagnosed and she was hospitalised in the coronary care unit for intravenous TT. Streptokinase 1 500 000 U was administered over 60 min in the fourth hour of chest pain. Two-dimensional echocardiographic examination at the bedside (Vivid 3 Pro) was performed following the therapy. It revealed left ventricular, left atrial and right-sided dilatations, third-degree mitral and second-degree tricuspid insufficiency, ejection fraction of 40 to 45% and 36 mmHg pulmonary artery pressure. No thrombus was detected in any cavity.

**Fig. 2.**
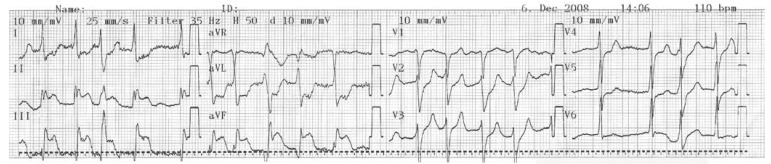
The second CT, 12 hours later, showing extensive infarction of the left frontal area.

The patient had deteriorated after two hours of intravenous TT. Motor aphasia and right hemiparesis then developed. The Babinski sign was positive in the right lower extremity. Immediate cerebral tomography (CT) was performed and showed no pathological abnormality. The patient then consulted a neurologist. In the second CT 12 hours later, extensive infarction of the left frontal area was seen (Fig. 2). On the follow up, neurological deficits had increased, congestive heart failure developed, and finally the patient died on the ninth day following TT.

## Discussion

STEMI is an urgent condition caused by acute thrombotic occlusion of the coronary arteries. Angiographic studies have shown that coronary arterial thrombosis is present in about 85% of patients with STEMI.^6^ The most important therapeutic method is immediate recanalisation of the infarct-related coronary artery. There are two methods to do this, PCI or a pharmacological approach.

PCI is the more favourable approach, but it presents many technical and scientific limitations. In our case, because of the respiratory insufficiency, the patient could not lie down so we could not do PCI. We decided to carry out intravenous TT with streptokinase because of its lower incidence of cerebral haemorrhage.

TT is an effective and easy therapeutic method that can be used anywhere and at any time. As in all therapeutic options, TT has certain limitations and complications. The most important and feared complication of a fibrinolytic agent is bleeding, especially intracranial bleeding. It occurs in 0.9% of patients treated with tPA.^5^ Bleeding after fibrinolytic treatment is due to the depletion of clotting factors and lysis of recently formed haemostatic plugs.^7^

TT for STEMI has reduced mortality at the expense of additional intracranial haemorrhage. The proof of efficacy of thrombolysis for STEMI comes from nine randomised placebocontrolled trials in a total of 58 511 patients. The meta-analysis of these trials showed an overall survival advantage of about 2% (11.5 vs 9.6%) in favour of thrombolysis.^7^ A meta-analysis of randomised trials comparing PCI with thrombolytics for STEMI at high-volume hospitals suggested that PCI improved 30-day survival free of reinfarction (11.9 vs 7.2%). Stroke risk was also reduced with PCI compared with thrombolytic therapy.^8^

Two randomised trials compared low-molecular weight heparin (LMWH), enoxaparin, with unfractionated heparin in patients with UA or non-STEMI.^9^,^10^ All patients received aspirin. In both studies, there were reductions in short-term outcomes of death, myocardial infarction (MI) and recurrent angina in patients randomised to LMWH. A combined analysis of these two trials showed significant 20% reductions in the short-term risk of death and non-fatal MI in patients randomised to LMWH. Randomised trials in STEMI patients conducted in the pre-fibrinolytic era showed that the risk of pulmonary embolism, stroke and re-infarction was reduced in patients who received intravenous heparin, providing support for the prescription of heparin to STEMI patients not treated with fibrinolytic therapy.

With the introduction of fibrinolytic therapy and, importantly, after the publication of the ISIS-2 trial,^3^ the situation became more complicated because of strong evidence of a substantial mortality reduction with aspirin alone, and confusing and conflicting data regarding the risk–benefit ratio of heparin used as an adjunct to aspirin or in combination with aspirin and a fibrinolytic agent. For every 1 000 patients treated with heparin compared with aspirin alone, there were five fewer deaths (p = 0.03) and three fewer recurrent infarctions (p = 0.04), at the expense of three major bleeds (p = 0.001).^11^

It is now clear that fibrinolysis recanalises thrombotic occlusions associated with STEMI, restores coronary flow, reduces infarct size and improves myocardial function and survival over both the short and long term.^1^,^12^,^13^ In patients receiving fibrinolysis for STEMI, the overall incidence of haemorrhagic complications is about 10%, and the incidence of intracranial haemorrhage is about 0.8%. Atrial fibrillation predisposes to atrial thrombus, and Crenshaw et al.^14^ demonstrated an increased risk for thrombotic strokes with atrial fibrillation in MI treated with TT.

In the present case, neurological signs were noted two hours after TT. It was assumed that the patient had had an intracranial haemorrhage. A cranial CT scan was immediately obtained, but haemorrhage was excluded and no abnormality was detected. In the second cranial CT scan, which was performed 12 hours later, a left frontal embolic infarction was detected. This is a rare condition. We continued to treat the STEMI and cerebral infarction, together with a neurologist/neurosurgeon consultant. On the follow up, the neurological problems progressed, congestive heart failure developed, clinical deterioration occurred, and subsequently the patient died.

In this case, although a thrombus was not detected during echocardiographic examination, it is likely that the TT induced lysis and fragmentation of an undetected microthrombus and the subsequent dislodging and embolisation of pre-existing cardiac microthrombi, which caused the cerebral infarction. Distal embolisation secondary to lysis of arterial thrombi in an aortic graft occlusion has been reported. It was speculated that this complication occurred when a combination of fresh and old thrombus was present.^15^ Rapid lysis of the fresh clot, along with arterial pulsations, may liberate older and more resistant clot fragments. When peripheral embolisation occurs, TT can be continued as long as the patient is clinically stable.

There is only one other case report in the literature describing embolic cerebral infarction following TT for STEMI.^1^

## Conclusion

This case represents an extremely rare clinical condition, which we report on to show the importance of the treatment of STEMI with TT. We deduced that the fact that the patient was in AF was a major contributing factor to her CVA. In conclusion, patients receiving TT for the treatment of STEMI should have constant neurological and cardiovascular re-evaluation and clinicians must be prepared to handle such complications in a timely manner.
